# How is hip anatomy reconstruction and inlay wear associated up to 10 years after primary THA using ceramic on highly crosslinked polyethylene bearings?

**DOI:** 10.1186/s12891-023-06501-y

**Published:** 2023-05-19

**Authors:** Johannes Weishorn, Samira Heid, Thomas Bruckner, Christian Merle, Tobias Renkawitz, Moritz M. Innmann

**Affiliations:** 1 Department of Orthopaedics, Heidelberg University Hospital, Schlierbacher Landstrasse 200a, 69118 Heidelberg, Germany; 2 Institute of Medical Biometry and Informatics, University of Heidelberg, Im Neuenheimer Feld 130.3, 69120 Heidelberg, Germany; 3 Department of Orthopaedic Surgery, Diakonie Klinikum Stuttgart, Stuttgart, Germany

**Keywords:** Cortical, Hypertrophy, Thigh, Pain, Short, Stem, Cementless, Hip, Arthroplasty, Hxlpe, Inlay, wear

## Abstract

**Introduction:**

Conventional polyethylene (PE) wear has been reported to be associated with femoral offset reconstruction and cup orientation after THA. Thus, the present study aimed (1) to determine the polyethylene wear rate of 32 mm ceramic heads with highly cross-linked polyethylene (HXLPE) inlays up to 10 years postoperatively and (2) to identify patient and surgery-related factors affecting the wear rate.

**Methods:**

A prospective cohort study was performed, investigating 101 patients with 101 cementless THAs and ceramic (32 mm) on HXLPE bearings after 6–24 months, 2–5 years and 5–10 years postoperatively. The linear wear rate was determined using a validated software (PolyWare®, Rev 8, Draftware Inc, North Webster, IN, USA) by two reviewers, blinded to each other. A linear regression model was used to identify patient and surgery-related factors on HXLPE -wear.

**Results:**

After an initial bedding-in phase of 1 year after surgery, the mean linear wear rate was 0.059 ± 0.031 mm/y at ten years (mean 7.7 years; SD 0.6 years, range 6–10), being below the osteolysis relevant threshold of 0.1 mm/year. The regression analysis demonstrated that age at surgery, BMI, cup inclination or anteversion and the UCLA score were not associated with the linear HXLPE-wear rate. Only increased femoral offset showed a significant correlation with an increased HXLPE-wear rate (correlation coefficient of 0.303; p = 0.003) with a moderate clinical effect size (Cohen’s f²=0.11).

**Conclusion:**

In contrast to conventional PE inlays, hip arthroplasty surgeons may be less concerned about osteolysis-related wear of the HXLPE if the femoral offset is slightly increased. This allows focusing on joint anatomy reconstruction, hip stability and leg length.

## Introduction

In primary total hip arthroplasty (THA) aseptic loosening is one of the most common reasons for revision [1]. In the past, PE wear of conventional and UHMWPE liners caused substantial osteolysis and aseptic implant loosening [2]. Several technical innovations have been made, finally resulting in second generation HXLPE inlays with significantly reduced wear rates [3]. As a result, polyethylene wear rates dropped below the potential osteolysis threshold of 0.1 mm/y in many patients, reducing the risk for developing osteolysis by 87%[3]. Therefore, ceramic on HXLPE is one of the most common bearing combinations in use for primary THAs in the United States and worldwide [1]. Several studies show excellent survival and linear wear rates in the short and midterm follow-up for first and second generation HXLPE inlays compared to UHMWPE [4, 5].

Several factors have been reported to be associated with the wear rate of PE inlays in primary cementless THA. On the one hand, individual patient related factors like gender, age, body mass index (BMI) and physical activity level may affect the PE wear rate [6, 7]. On the other hand, individual surgical factors like combined cup and stem orientation, reconstruction of the position of the center of rotation, including height and offset may affect the PE wear rate [8, 9]. Particularly, femoral offset reconstruction seems to be a relevant factor. Little et al. found an increased wear rate of 0.16 mm/year for UHMWPE liner in patients with increased femoral offset of more than 5 mm after THA compared to preoperative findings [10]. This finding is of clinical relevance for hip arthroplasty surgeons. It has been demonstrated, that hip anatomy reconstruction, specifically leg length and offset, and implant orientation affect joint stability, gait pattern and patient reported outcome [6, 11]. Depending on the surgeon’s preferred acetabular reaming technique, individual acetabular bone stock or anatomic deformity, the center of rotation is often slightly medialized in order to achieving sufficient press-fit for primary stability of cementless cups [12, 13]. Consequently, the femoral offset has to be increased, in some cases by more than 5 mm, compensating for the loss of acetabular offset, in order to achieving joint stability without excessive leg lengthening. For UHMWPE liners, hip arthroplasty surgeons had to make a trade-offs, weighing the risk of hip instability against increased PE wear, potentially negatively affecting long term survival due to aseptic implant loosening. However, no association has been reported between hip anatomy reconstruction and liner wear rate of HXLPE liners, the development of osteolysis or aseptic loosening.

Therefore, the present study aimed (1) to determine the polyethylene wear rate of bearings of 32 mm ceramic heads with HXLPE inlays up to 10 years postoperatively and (2) to identify patient and surgery-related factors affecting the wear rate of HXLPE inlays.

## Materials and methods

### Study cohort

The present study investigated a cohort of 101 consecutive patients with 101 THAs. All patients received a THA with HXLPE inlays and ceramic heads with a diameter of 32 mm. Data was collected prospectively with an institutional database. The aims, design, inclusion / exclusion criteria and statistical analysis were defined before starting the study. A sample size calculation was performed a priori with GPower® (G’*Power Version 3.1.9.7; University of Duesseldorf, Duesseldorf, Germany) indicating that a cohort of 96 hips would be needed to answer the study question with sufficient power (0.8), assuming a wear rate of 0.02 mm for HXLPE, as described by the manufacturer. The interpretation of the power analysis was based on an accuracy of the Devane wear measurement method of +/- 0.076 mm for the PolyWare software [14]. The study was approved by the institutional review board (S—083/2017) and performed in accordance with the current version of the Declaration of Helsinki 2013.

**Fig. 1:**
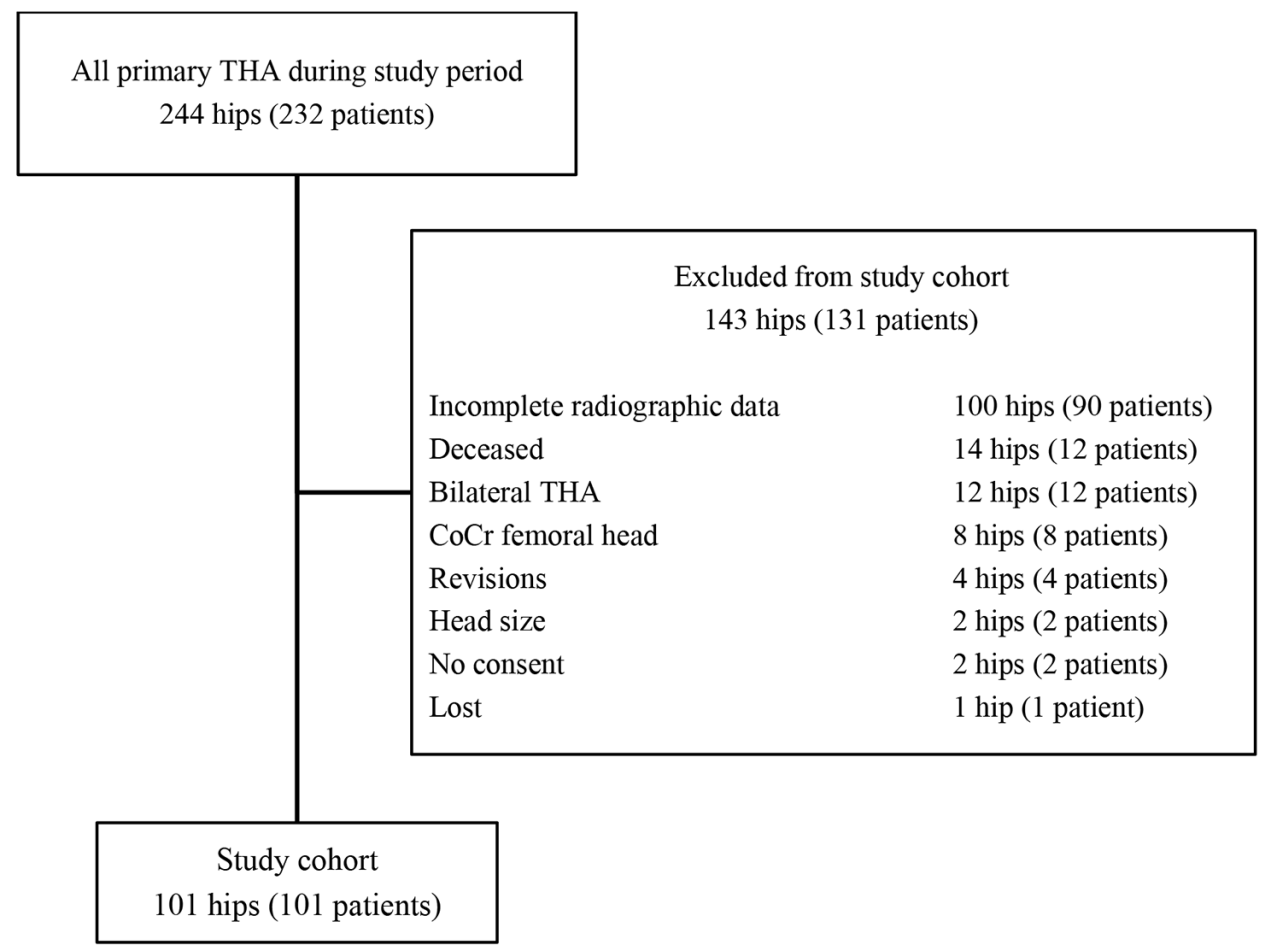
Identification of the study cohort

During the study period between December 2007 and 2009, a total of 836 primary THAs were performed at our institution. 244 THAs in 232 patients met the inclusion criteria of advanced primary osteoarthritis of the hip, avascular necrosis of the femoral head, mild developmental dysplasia of the hip Crowe grade I (lateral center-edge angle of 20–25°) and rheumatoid arthritis, using a curved, cementless, bone preserving, meta-diaphyseal anchoring stem (Fitmore®) and cementless, pressfit acetabular cup design (Allofit®), manufacturer Zimmer, Warsaw, IN, USA. As bearing surface remelted, first generation HXLPE inlays (Durasul®, Zimmer, Warsaw, IN, USA) were used with 32 mm diameter ceramic heads with 3 different neck length options in all patients (-4;0;4 mm; Biolox®, CeramTech, Plochingen, GER). An anterolateral Watson-Jones or lateral Bauer approach was utilized in all patients. Demographics are given in Table 1. Radiographs were taken directly pre- and postoperatively, at 6–24 months, at 2–5 years and at 5–10 years postoperatively. After minimum follow-up of 5 years 101 hips in 101 patients were left for evaluation. Patients with incomplete radiographic data sets, bilateral THAs, withdrawn consent, revised THAs or death before a minimum follow-up of 5 years were excluded (Fig. 1). A subgroup analysis was performed comparing radiographic and clinical results between patients with a linear wear rate below and above 0.1 mm/y, which has been decribed as osteolysis threshold in the literature [3].

**Table 1:** Patient’s demographics and diagnosis stratified by the linear wear rate threshold of 0.1 mm/y

Variable	all	> 0.1 mm/y	< 0.1 mm/y	p
Demographics				
Number of hips	101	9	92	
Gender (m : w)	55:46	3:6	52:40	
Age at surgery (y)	58.0 (11.4)	63.2 (6.6)	57.4 (11.8)	0.15
BMI (kg/m²)	26.5 (4.5)	23.7 (2.8)	26.8 (4.5)	0.036*
**Diagnosis**				
Primary osteoarthritis	54	6	48	
Developmental dysplasia	25	2	23	
Avascular necrosis	7	1	6	
Posttraumatic osteoarthritis	4	0	4	
Rheumatoid arthritis	6	0	6	
Others	5	0	5	

* indicating significance (p < 0.05); mean values (SD)

### Clinical and radiographic assessment

**Fig. 2:**
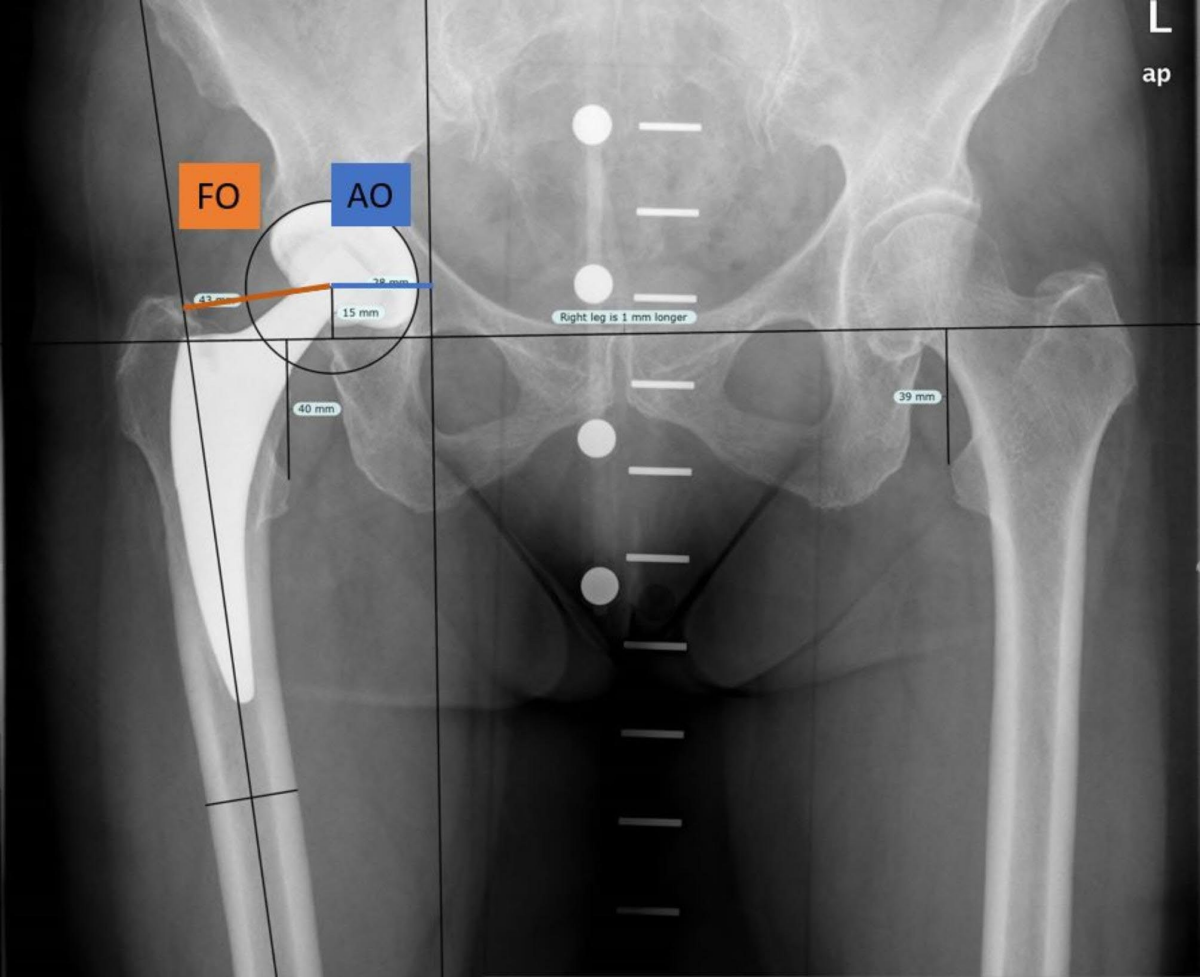
Illustration of radiographic offset measurements

Clinical and radiographic outcome was assessed after 1, 2 to 5 and 5 to 10 years postoperatively. Digital antero-posterior (ap) radiographs of the pelvis, with the radiation beam centered on the pubic symphysis and 15° internally rotated legs, and lateral Lauenstein radiographs were taken at each follow up in a standardized technique. Patient reported outcome and functional scores were assessed using validated questionnaires at each visit (WOMAC [15], SF-36 [16], UCLA [17] and Tegner [18]). Radiographic measurements and wear analyses were performed by two independent reviewers, blinded to each other. Peri-implant radiolucencies and osteolysis were determined on ap-pelvis radiographs and grouped by zones described by Gruen et al. [19] Measurements for hip reconstruction parameters, in particular stem alignment, acetabular offset (AO) and femoral offset (FO), were performed on calibrated postoperative ap-radiographs of the pelvis using ImageJ software v1.44 (National Institute of Health, USA) and Roman software v1.70 (Institute of Orthopedics, Owestry, UK). As described previously, the FO was measured as the perpendicular distance between the femoral axis and the center of rotation of the femoral head (COR) [20–22]. The AO was determined as the distance between the COR and a vertical line through the ipsilateral teardrop Fig. [21]. Hip offset (HO) was calculated as the sum of FO and AO (Fig. 2). Radiographic leg length (LL) was measured as distance between the trans-teardrop line (TTL) and the lesser trochanter on each side [21]. Leg length difference (LLD) was calculated as the difference between leg length of both hips.

**Fig. 3:**
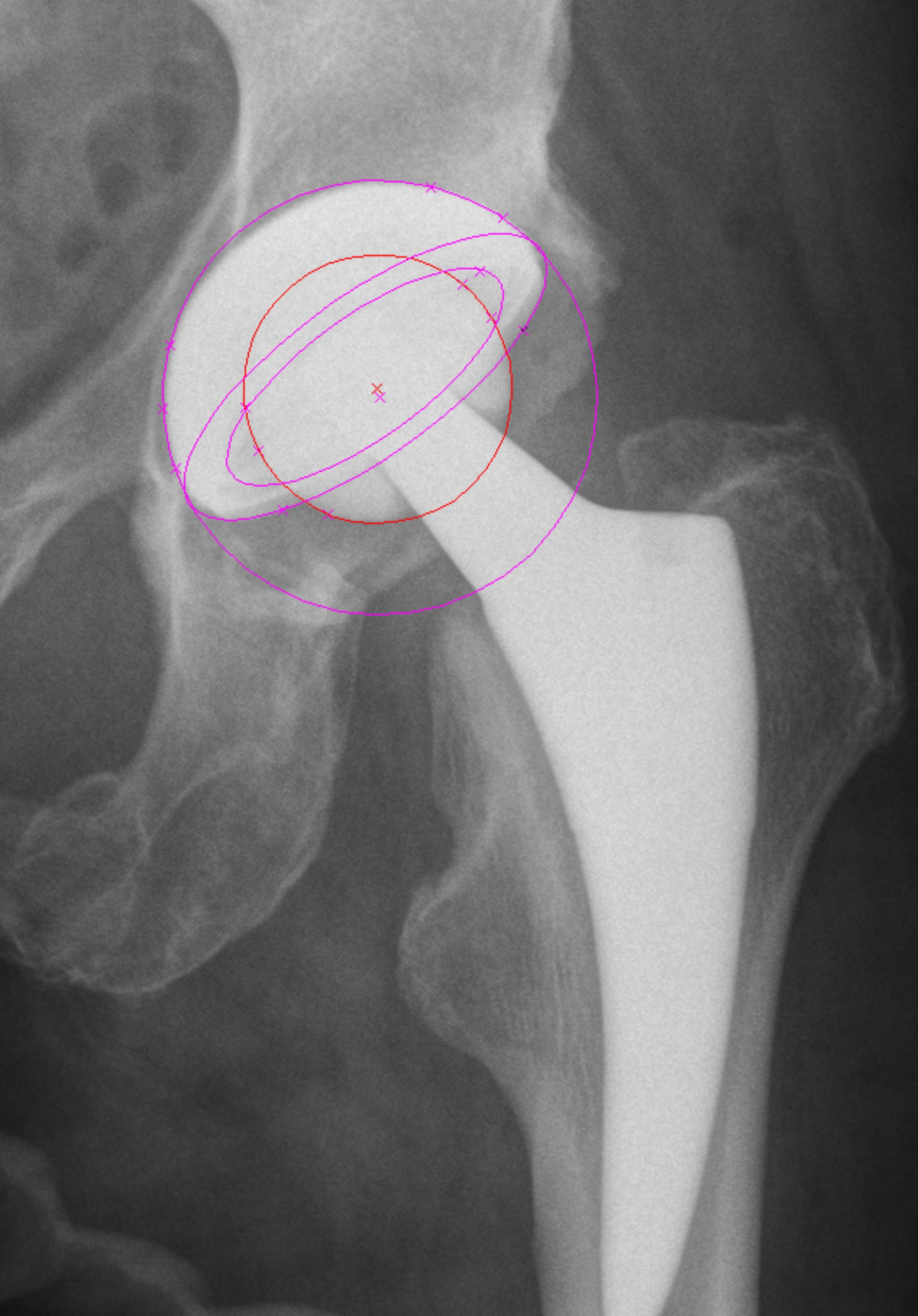
Wear measurements using PolyWare® shown in ap-Radiographs

Measurements for cup orientation (inclination and anteversion) and wear analyses were performed using validated software PolyWare (PolyWare®, Rev 8, Draftware Inc, North Webster, IN, USA) based on Devanes’ method as published before (Figs. 2 and 3) [23]. Linear wear was determined between 1 year and last FU, accounting for an initial bedding-in period in the first year after surgery. Inter- and intra-observer reliability analyses were conducted twice with 40 randomly selected hips (40%) showing good to excellent values between 0.74 (0.7–0.78) and 0.91 (95%-KI; 0.86–0.95).

### Statistics

The sample size calculation was performed a priori as described above. An additional post hoc power analysis was performed for the multivariate regression model showing an excellent power of 0.9 (1- β error) (6 predictors, R²=0.078, sample size of 101 hips, α-error of 0.05, effect size of 0.085). Potential factors being associated with the HXLPE wear rate were analyzed using linear correlation analyses (Pearson’s correlation coefficient for parametric and Spearman’s rank correlation coefficient for non-parametric variables), bivariate regression analysis in case of a non-linear dependency, and a multivariate regression model. The level of significance was p ≤ 0.05 for all tests. SPSS® Version 25 (IBM SPSS Statistics, IBM, Armonk, NY, USA) was utilized for analyzing the data.

## Results

### Radiographic outcome

No aseptic loosening or osteolysis was observed in the studied cohort. Mean cup inclination and anteversion was 41.7° (41.7 ± 7.1) and 23.6° (23.6 ± 8.5), respectively. On average, acetabular offset was decreased by 7.4 mm (-7.4 ± 6.2) and femoral offset increased by 5.9 mm (5.9 ± 6.7) compared to preoperatively, resulting in a slightly decreased overall hip offset of -1.5 mm (-1.5 ± 6.3). Radiographic measurements are given in Table 2.

### Linear wear rate

After a mean follow up of 7.7 years (SD 0.6, range 6–10 years) the mean linear wear rate was 0.059 mm per year (SD 0.031 mm/y). The univariate correlation analyses demonstrated, that femoral and acetabular offset reconstruction were the only radiographic parameters correlating with the linear wear rate.

**Table 2:** Univariate linear regression analysis – dependent variable linear wear rate

N = 101	Value, mean (SD)	Adjusted R²	Adjusted Coefficient ß		p-value
Age at surgery (y)	58.0 (11)	0.001	0.1		0.31
BMI (kg/m²)	26.5 (4.5)	0.018	-0.17		0.1
UCLA (points)	6.3 (1.9)	-0.009	0.03		0.74
Stem alignment (°)	2.9 (2.6)	0.023	-0.18		0.07
Cup size (mm)	53.1 (3.5)	-0.009	0.04		0.71
Inclination (°)	41.7 (7.1)	0.002	-0.11		0.28
Anteversion (°)	23.6 (8.5)	0.002	0.11		0.28
Δ LLD (mm)	5.1 (5.9)	0.004	0.12		0.25
ΔFO (mm)	5.9 (6.7)	0.078	0.3		0.003*
ΔAO (mm)	-7.4 (6.2)	0.030	-0.2		0.046*
ΔHO (mm)	-1.5 (6.3)	0.004	0.12		0.24

* indicating significance (p < 0.05); mean values (SD)

### Subgroup analysis by osteolysis threshold

**Fig. 4:**
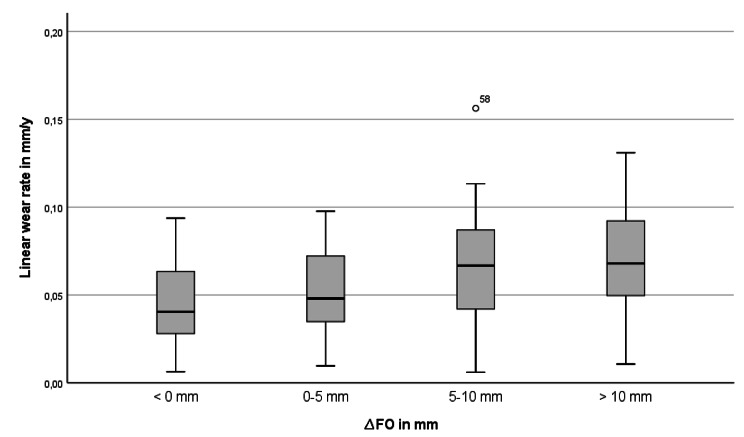
Boxplots for the linear wear rate stratified for femoral offset groups,demonstrating slightly increased wear with increasing femoral offset

9 of 101 patients (9%) showed wear rates above the osteolysis threshold of 0.1 mm/y. Compared to patients with wear rates below the threshold, patients with wear rates above the threshold showed a decreased BMI (23.7 ± 2.8 vs. 26.8 ± 4.5; p = 0.036), an increased postoperative femoral offset (12.4 ± 4.4 mm vs. 5.2 ± 6.6 mm; p = 0.002) and increased hip offset (2.9 ± 4.6 mm vs. -1.9 ± 6.4 mm; p = 0.029 ). The linear wear rate, stratified for femoral offset groups is given in Fig. 4. No difference could be found between both groups for cup inclination (37.9 ± 8.7 vs. 42.0 ± 6.9; p = 0.095), anteversion (26.6 ± 8.1 vs. 23.3 ± 8.6; p = 0.28), stem axis (3.1 ± 1.9 vs. 2.9 ± 2.7; p = 0.73), leg length difference (6.3 ± 5.3 vs. 5.0 ± 5.9; p = 0.51) patient reported outcome, physical activity level, age or gender. (Tables 1 and 3)

**Table 3:** Clinical Outcome and Radiographic measurement

Variable	Value, mean (SD)	> 0,1 mm/y threshold	< 0,1 mm/y threshold	p
Patient reported outcome				
HHS, mean (SD)	92.8 (12.3)	90.6 (15.0)	93.0 (12.2)	0.79
Tegner, mean (SD)	3.6 (1.3)	3.8 (1.5)	3.6 (1.3)	0.84
UCLA, mean (SD)	6.3 (1.9)	6.2 (1.6)	6.3 (2.0)	0.9
**Radiographic Assessment**				
Stem alignment (°)	2.9 (2.6)	3.1 (1.9)	2.9 (2.7)	0.73
Cup inclination (°)	41.7 (7.1)	37.9 (8.7)	42.0 (6.9)	0.095
Cup anteversion (°)	23.6 (8.5)	26.6 (8.1)	23.3 (8.6)	0.278
∆LLD (mm)	5.1 (5.9)	6.3 (5.3)	5.0 (5.9)	0.51
∆AO (mm)	-7.4 (6.2)	-7.2 (5.4)	-9.5(6.3)	0.29
∆FO (mm)	5.9 (6.7)	12.4 (4.4)	5.2 (6.6)	0.002**
∆HO (mm)	-1.5 (6.3)	2.9 (4,6)	-1.9 (6.4)	0.029*
Linear wear rate (mm/y)	0.059(0.031)	0.120 (0.016)	0.053 (0.025)	0.000***

* indicating significance *(p < 0.05) **(p < 0.01) ***(p < 0.001); mean values (SD)

### Multivariate linear regression analysis

The multivariate linear regression analysis demonstrated that age at surgery, BMI, cup inclination, cup anteversion and the UCLA Score were not associated with linear wear rate of HXLPE inlays (p > 0.05). Only the postoperative change in femoral offset showed an association with the linear wear rate of HXLPE inlays (p = 0.002) (Table 4). However, the clinical effect size was moderate (Cohen’s f² =0.11).

**Table 4:** Multivariate linear regression analysis – dependent variable linear wear rate (R²=0.1)

N = 101	Value, mean (SD)	Adjusted Coefficient ß	p-value
Age at surgery (y)	58.0 (11)	0.163	0.116
BMI (kg/m²)	26.5 (4.5)	-0.111	0.268
UCLA (points)	6.3 (1.9)	0.048	0.645
Inclination (°)	41.7 (7.1)	-0.080	0.418
Anteversion (°)	23.6 (8.5)	0.092	0.354
Δ LLD (mm)	5.1 (5.9)	0.131	0.187
ΔFO (mm)	5.9 (6.7)	0.303	0.002^*^

* indicating significance (p < 0.05); mean values (SD)

## Discussion

A prospective cohort study was conducted to evaluate ceramic (32 mm) on HXLPE bearings in THA. After an initial bedding-in period of 1 year post-operatively, the mean linear wear rate was 0.059 ± 0.031 mm/year at 8 years, which is below the osteolysis relevant threshold of 0.1 mm/year.

Osteolysis and aseptic loosening has been a matter of great concern for many years in cementless THA using conventional PE inlays, especially in young and active patients. The following developments of cups, stems, heads and particularly PE inlays have dramatically improved the long-term wear-associated survival rates, resulting in another success story of hip arthroplasty surgery [24].

However, this success did not result in complacency of hip arthroplasty surgeons and researchers. Other factors affecting implant survival and patient reported outcome have been increasingly investigated. Several studies could demonstrate, that implant orientation, offset and leg-length reconstruction affect the range of motion after THA, the risk of impingement or dislocation, patient reported outcome measures and the normalization of gait patterns [11, 25, 26]. The association of both research topics – inlay wear and the reconstruction of hip anatomy parameters – has been investigated in only few studies. One of the best known studies by Little et al. demonstrated that increased femoral offset was associated with increased wear of UHMWPE inlays [27]. However, no such study has been performed for one of the most commonly used bearings – ceramic heads with a diameter of 32 mm and HXLPE inlays. In the present study we were able to demonstrate, that an increase in femoral offset significantly correlated with the HXLPE-wear rate up to 10 years postoperatively, similar to reports for UHMWPE inlays [27]. However in contrast to UHMWPE inlays and despite high significance, the association between femoral offset and HXLPE inlay wear was rather small. Only a small percentage (9%) of THAs showed linear wear rates slightly above the osteolysis threshold of 0.1 mm/y and no osteolysis or aseptic implant loosening could be found, resulting in a limited clinical relevance of the observed association after 5 to 10 years of follow up.

In the literature the difference in linear and volumetric wear rate between conventional UHMWPE-liner and HXLPE-liners is well described. For first generation HXLPE-inlays linear wear rates of 0.005 to 0.09 mm/y have been reported, which is well below the osteolysis threshold, while conventional UHMWPE-inlays exceed this value more frequently (0.038 to 0.41 mm/y) [5, 28–30]. For the HXLPE inlay, studied in the present cohort, an annually linear wear of 0.005 to 0.054 mm has been reported, which is in accordance with our present findings (0.059 ± 0.031 mm/y) [29, 31]. As a consequence of decreased PE wear, lower incidence of osteolysis, aseptic loosening and longer implant survival could be demonstrated [2, 4]. A randomized controlled trial detected 9% vs. 46% of osteolysis and no vs. 12% of reoperations for wear-related complications 15 years after THA in patients with HXLPE vs. UHMWPE liners [2]. Similarly, Devane et al. reported a prevalence of 8% vs. 38% for osteolysis 10 years after THA (HXLPE vs. UHMWPE)[4]. For UHMWPE liners, several factors could be identified that are associated with an increased inlay wear rate [10]. Little et al. could demonstrate reduced UHMWPE wear for THAs with reconstructed femoral offset within 5 mm of the native femoral offset (p = 0.0094) and cup inclination angles below 45° (p = 0.012) [10]. Furthermore Devane et al. investigated the influence of offset reconstruction and wear rate in UHMWPE liners and concluded that under-restoration of femoral offset could also lead to an increase in PE wear [32]. Consequently, the target range for offset reconstruction in order to minimize wear appeared to be small for UHMWPE inlays.

In the literature, only a single prospective cohort study investigated potential factors being associated with the wear rate of HXLPE inlays [33]. Cheung et al. could find no association of femoral offset reconstruction and the linear inlay wear rate (p = 0.651, r=-0.17), but as the study investigated a cohort of 87 patients, it might have lacked sufficient statistical power in order to detect significance [33]. In the same article, they found significant, but weak correlations between cup orientation parameters (inclination (r = 0.256, p = 0.014 and r = 0.221, p = 0.035, respectively) and the linear HXLPE wear rate [33]. In our present study, we could find no association between acetabular reconstruction parameters and the wear rate of HXLPE inlays in the multivariate model, which is supported by several other studies for HXLPE inlays, demonstrating also no association of cup orientation parameters and the linear wear rate [8, 9, 34]. Studies investigating the effect of femoral head size and material on polyethylene wear rates of HXLPE inlays show controversial results [34, 35]. Small differences for PE wear between 32 and 36 mm diameter heads did not reach statistical significance and remained under the osteolysis threshold for both groups after 5 years [34]. Similarly, no significant difference could be found cobalt-chromium and ceramic heads after 5 years [34]. Thus, the assumption that polyethylene wear would increase with femoral head size seems not to be of clinical relevance in modern C-O-HXLPE or M-O-HXLPE bearings, at least after 5 years [3, 35]. As the present study investigated 101 THAs, all with same bearing consisting of 32 mm diameter ceramic head articulating with a HXLPE inlay, we could consequently not report on potential associations between head size or material and the wear rate, which is a limitation of the study.

This study has further limitations. Several patients having received THA during the study period did not meet the inclusion criteria and had to be excluded. However, in order to answer the study questions, a homogeneous study cohort had to be investigated, requiring to excluding patients with incomplete radiographic follow-up, contralateral THAs, non-wear-related revisions, and implant heads other than 32 mm ceramic, which might have introduced a potential selection bias. Due to the strict inclusion and exclusion criteria, an a priori power analysis was conducted, indicating a sufficient sample size in order to answering our study questions [28, 33]. However, the study might have still been underpowered, because the accuracy of the wear measurement method of +/- 0.076 mm, which was used for the power calculation, was from a phantom study that might have had optimized laboratory conditions potentially leading to a accuracy as in a clinical setting, potentially leading to an underestimation of detectable wear in the present clinical setting. Furthermore, wear measurements were performed using ap and lateral radiographs in order to determine the linear PE wear rate. Measuring true PE wear would be a three-dimensional process requiring using CT-scans, what was not approved by our institutional review board. This might have resulted in a certain inaccuracy of our wear measurements. However, the present way of measuring and reporting PE wear has been published in several studies, allowing to interpreting our results in the context of the literature. Furthermore, measured head penetration rate is a combination of true abrasive wear and plastic deformation. We considered the first 24 months as initial bedding phase, but in few cases bedding in has been reported to take place up to 3 years post THA [34]. Therefore, our measurements might overestimate the linear wear rate of the HXLPE liners to some degree. Femoral offset measurements might have been underestimated due to rotational errors of the femur as reported in the literature [20, 36]. However, the pre-/postoperative change in femoral offset was investigated and not the absolute values for FO. Thus, there is a low risk of a systematic bias for the correlation and regression analysis. Lastly, we did not have stem anteversion measurements. Therefore, a potential association of combined acetabular and femoral version with the polyethylene wear rate could not be detected.

### Conclusion

In conclusion, cementless THAs with highly cross-linked PE inlays and 32 mm diameter ceramic heads showed a low mean linear wear rate of 0.059 mm/year without osteolysis or aseptic loosening. Demographics, physical activity, implant orientation and hip geometry reconstruction did not clinically relevantly affect the linear wear rate of HXLPE inlays up to 10 years after THA. Hip arthroplasty surgeons may be concerned much less about inlay wear with HXLPE than previously with UHMWPE inlays, allowing to focus on hip anatomy reconstruction, in order to achieve good range of motion, joint stability, minimized leg length difference and optimum patient satisfaction after THA. Furthermore, the investigated inlays are first generation HXLPE inlays. Given the present results with low wear rates, these inlays are still a viable option compared to other, newer generation HXLPE inlays.

## Data Availability

The datasets used and/or analysed during the current study is available from the corresponding author (Moritz M. Innmann) on reasonable request.

## Abbreviations

PE: Polyethylene
HXLPE: Highly crosslinked polyethylene
mm: Millimeter
y: Year
SD: Standard deviation
BMI: Body mass index
UCLA: University of California, Los Angeles
THA: Total hip arthroplasty
UHMWPE: Ultra-High Molecular Weight Polyethylene
AO: Acetabular offset
FO: Femoral offset
COR: Center of rotation
HO: Hip offset
LL: Leg length
TTL: Trans-teardrop line
LLD: Leg length difference
FU: Follow up
C-O-HXLPE: Ceramic on highly crosslinked polyethylene
M-O-HXLPE: Metal on highly crosslinked polyethylene
